# ZAR1 is a novel epigenetically inactivated tumour suppressor in lung cancer

**DOI:** 10.1186/s13148-017-0360-4

**Published:** 2017-06-02

**Authors:** Antje M. Richter, Steffen Kiehl, Nicole Köger, Janina Breuer, Thorsten Stiewe, Reinhard H. Dammann

**Affiliations:** 1 Institute for Genetics, Justus-Liebig-University Giessen, 35392 Giessen, Germany; 2 Institute of Molecular Oncology, Philipps-University Marburg, 35043 Marburg, Germany; 3 German Center for Lung Research (DZL), Universities of Giessen and Marburg Lung Center, 35392 Giessen, Germany

**Keywords:** Lung cancer, ZAR1 (zygote arrest 1), Tumour suppressor, DNA methylation, Epigenetics

## Abstract

**Background:**

Lung cancer is the leading cause of cancer-related deaths with 1.8 million new cases each year and poor 5-year prognosis. Promoter hypermethylation of tumour suppressors leads to their inactivation and thereby can promote cancer development and progression.

**Results:**

In this study, we analysed ZAR1 (zygote arrest 1), which has been said to be a maternal-effect gene and its expression mostly limited to certain reproductive tissues. Our study shows that *ZAR1* is expressed in normal lung but inactivated by promoter methylation in lung cancer. *ZAR1* is hypermethylated in primary lung cancer samples (22% small cell lung carcinoma (SCLC) and 76% non-small cell lung carcinoma (NSCLC), *p* < 0.001) vs. normal control lung tissue (11%). In lung cancer cell lines, *ZAR1* was significantly methylated in 75% of SCLC and 83% of NSCLC vs. normal tissue (*p* < 0.005/0.05). In matching tumours and control tissues, we observed that NSCLC primary tumour samples exhibited a tumour-specific promoter methylation of *ZAR1* in comparison to the normal control lung tissue. Demethylation treatment of various lung cancer cell lines reversed *ZAR1* promoter hypermethylation and subsequently re-established *ZAR1* expression. In addition, we could show the growth inhibitory potential of ZAR1 in lung cancer cell lines and cancer cell lines. Exogenous expression of *ZAR1* not only inhibited colony formation but also blocked cell cycle progression of cancer cell lines.

**Conclusions:**

Our study shows for the first time the lung tumour-specific epigenetic inactivation of *ZAR1* due to DNA methylation of its CpG island promoter. Furthermore, ZAR1 was characterised by the ability to block tumour growth through the inhibition of cell cycle progression in cancer cell lines. We propose that ZAR1 could serve as an epigenetically inactivated biomarker in lung cancer.

**Electronic supplementary material:**

The online version of this article (doi:10.1186/s13148-017-0360-4) contains supplementary material, which is available to authorized users.

## Background

Lung cancer is currently the leading cause of cancer related deaths in men and the second leading cause of cancer death in women worldwide [1]. Lung cancer has a high incidence coupled with a poor 5-year survival rate of less than 17% [2]. In 2012, 1.8 million cases were newly diagnosed worldwide and 1.6 million deaths from lung cancer occurred [3]. Tobacco smoking still is the main cause for lung cancer development [4]. Lung cancer can be classified histologically as small cell lung carcinoma (SCLC, 20% of cases) and non-small cell lung carcinoma (NSCLC, 80% of cases), and NSCLC can further be divided into adenocarcinoma, squamous cell carcinoma, and large cell carcinoma subtypes [5]. Lung cancer treatment as well as prognosis differ depending on lung cancer subtypes [6]. There are a plethora of known lung cancer associated genomic and epigenetic alterations, and it was summarised that epigenetic modulators, modifiers, and mediators play a crucial role in lung cancer initiation and progression [5, 7].

Our own experiments regarding lung cancer analysed epigenetic alterations in the lung cancer cell line A549 using the Infinium HumanMethylation450K BeadChip Array (Illumina). We found the maternal effect gene *ZAR1* was one of the best candidates [8]. Human *ZAR1* or zygote arrest 1 is located on chromosome 4 (4p11) and covered by a 1.5 kb large CpG island (Additional file 1: Figure S1). CpG islands are genomic regions defined by the enrichment of CpG dinucleotides [9]. *ZAR1*’s 1275 nt transcript from 4 exons encodes 424 amino acid proteins containing a C-terminal zinc finger (CpG plot, NCBI and UCSC genome browser).

ZAR1 was described as a novel maternal-effect gene critical for oocyte to embryo transition in mouse and especially the C-terminus of its protein is evolutionarily highly conserved in vertebrates [10, 11], but later studies found that its expression was not limited to the oocyte [12–14].

Regarding an association of human ZAR1 and cancer only, few reports exist. In malignant melanoma, *ZAR1* was intra-genically methylated (exon 1) and ZAR1 was overexpressed in some hypermethylated melanoma cell lines [15]. Non-promoter hypermethylation was found in brain tumours and neuroblastoma [16, 17]. Expression of *ZAR1* was absent in hypermethylated glioma cell lines [16], but *ZAR1* was detected in hypermethylated neuroblastoma [17]. It was proposed that methylation-related aberrant ZAR1 expression was unlikely to be related to glioma tumorigenesis [16]. *ZAR1* intragenic methylation (first exon to intron 1) in sporadic bladder cancer was decreased in high-grade vs. low-grade tumours [18]. In hepatitits C virus, positive hepatocellular carcinoma *ZAR1* hypermethylation of exon 1 was found [19]. These previous data concentrated on non-promoter methylation and *ZAR1* expression levels were inconsistent.

We hypothesised that ZAR is a tumour suppressor inactivated in cancer by its promoter hypermethylation. In our study, we found that *ZAR1* is epigenetically inactivated in lung cancer. We further found evidence that ZAR1 acts as a tumour suppressor and therefore suggest it could serve as a biomarker in lung cancer cells.

## Results

### ZAR1 is epigenetically inactivated in lung cancer

**Fig. 1:**
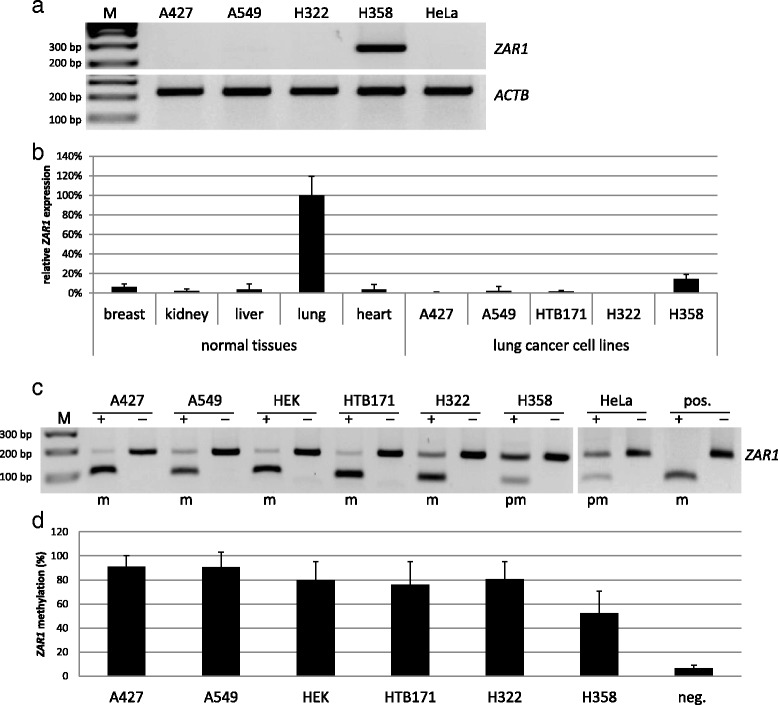
*ZAR1* expression pattern, missing expression in lung cancer and promoter hypermethylation in lung cancer cell lines. *ZAR1* expression in lung cancer cell lines A427, A549, HTB171, H322, H358 and HeLa (cervix carcinoma) versus normal tissue panel (breast, kidney, liver, lung and heart) by RT-PCR and semi-/quantitative PCR were determined. **a**, **b** Expression levels were normalised to *ß-ACTIN* (*ACTB*) and to normal lung tissue (=100%). Promoter hypermethylation of lung cancer cell lines, HEK and HeLa cells by COBRA analysis **c** and pyrosequencing for quantification **d** are shown. PCR and digestion products were separated on 2% TBE agarose gel with 100 bp marker *M* (*m* methylated, *pm* partially methylated, *pos.* positive control, *neg.* negative control; + *TaqI* digested, − mock digested)

In an Infinium HumanMethylation450K BeadChip Array (Illumina), we observed altered promoter methylation of the lung cancer cell line A549 [8]. *ZAR1* (zygote arrest 1) was amongst the most strongly deregulated genes, and up to date, no study investigated the epigenetic inactivation of the *ZAR1* promoter and its consequences in lung cancer. We found that *ZAR1* is expressed in normal lung (Fig. 1b), but its expression is lost in lung cancer cell lines (Fig. 1a, b). Studying the genomic organisation of *ZAR1*, we found a CpG island of 1.5 kb, which covered the promoter and its first exon (Additional file 1: Figure S1). ZAR1 is encoded by four exons, it contains 424 aa and an uncharacteristic C-terminal zinc finger. In ZAR1’s structure, we could not find an NLS signal and in accordance, it was said that ZAR’s zinc finger possibly facilitates RNA binding [20].

**Fig. 2:**
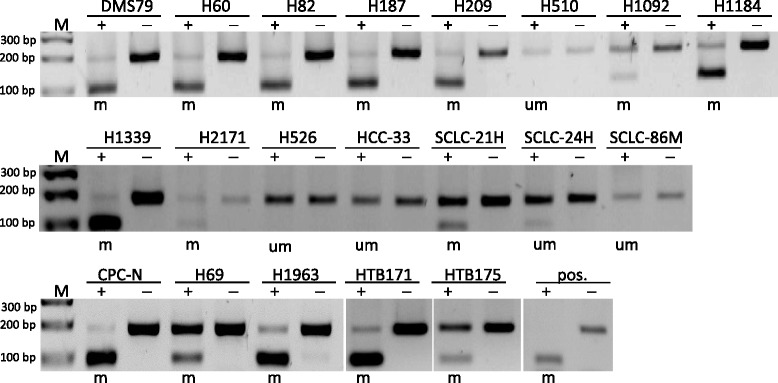
*ZAR1* promoter hypermethylation in lung cancer cell lines (SCLC). *ZAR1* promoter methylation status was analysed by COBRA assay in various SCLC lung cancer cell lines. CpG island region from the *ZAR1* promoter was amplified from bisulfite-treated DNA and digested with *Taq*1. Digestion products indicate *ZAR1* promoter methylation. Digestion products were separated on 2% TBE agarose gel with 100 bp marker (*m* methylated, *um* unmethylated, *pos.* positive control, + *TaqI*-digested, − mock digested)

**Fig. 3:**
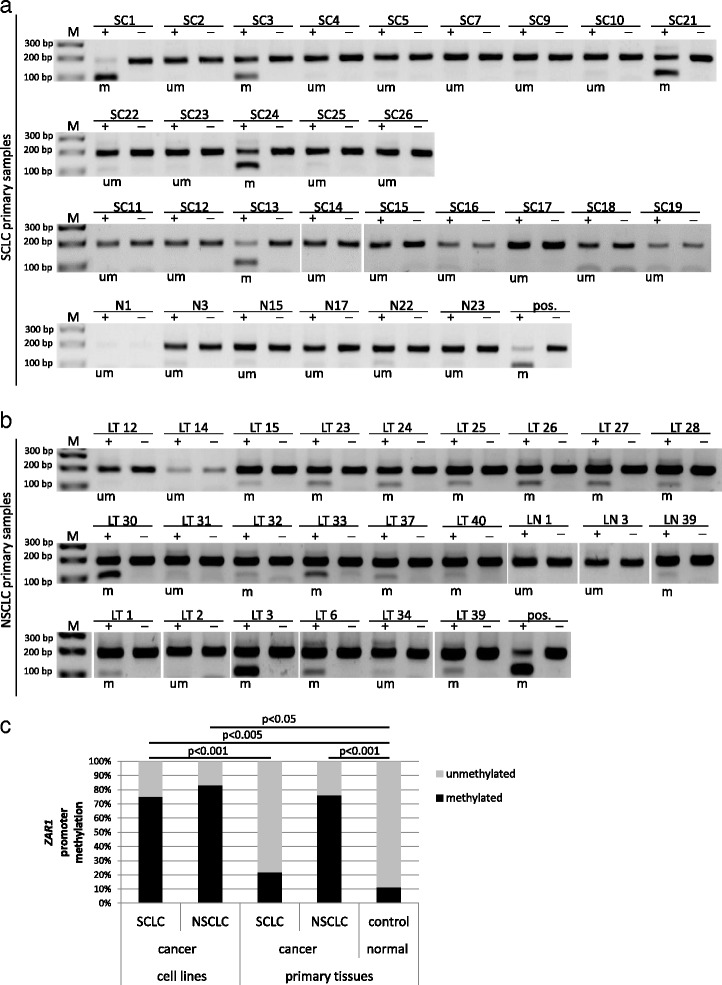
*ZAR1* promoter hypermethylation in primary lung tumours and summary of ZAR1 methylation. *ZAR1* promoter methylation was studied by COBRA assay in primary lung tumours of SCLC and NSCLC samples and in normal control tissue. SCLC tumour samples were abbreviated SC and control tissue N (**a**). NSCLC tumour samples were abbreviated LT (lung tumours) and control tissue LN. LN1, LN3 and LN39 were matching samples to tumour samples LT1, LT3 and LT39 (**b**). CpG island region from the *ZAR1* promoter was amplified from bisulfite-treated DNA and digested with *Taq*I. Digestion products indicate *ZAR1* promoter methylation. Digestion products were separated on 2% TBE agarose gel with 100 bp marker (*m* methylated, *um* unmethylated, *pos.* positive control, + *TaqI*-digested, − mock digested). **c** Summary of *ZAR1* promoter methylation in lung cancer cell lines and primary lung tumours is shown (Fisher exact test)

**Fig. 4:**
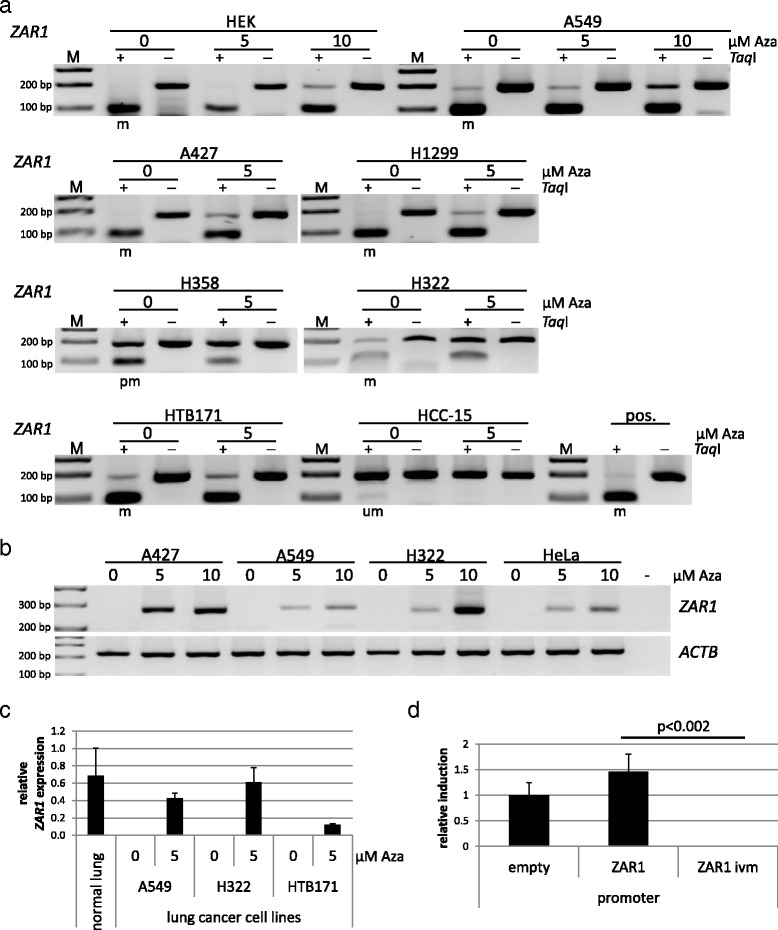
Reversal of *ZAR1* epigenetic inactivation in cancer cells by demethylation treatment. Various lung cancer cell lines as well as HEK and HeLa cells were treated with the demethylating agent 5-Aza-2’-deoxycytidine (0, 5 or 10 μM Aza). COBRA assay under Aza treatment shows demethylation of the *ZAR1* promoter (**a**) and correlating reexpression of *ZAR1* levels by semi-quantitative RT-PCR (**b**). PCR and digestion products were separated on 2% TBE agarose gel with 100 bp marker (*m* methylated, *pm* partially methylated, *um* unmethylated, *pos.* positive control, + *TaqI-*digested, − mock digested). **c** Real-time PCR quantification of *ZAR1* reexpression after 5 μM Aza treatment, which was normalised to *GAPDH*. **d** Luciferase reporter assay was performed to determine the *ZAR1* promoter activity. The artificial *ZAR1* promoter was cloned into pRLnull and subsequently in vitro methylated. The promoters were transfected in HeLa cells and the *ivm ZAR1* promoter showed full inactivation in contrast to its unmethylated construct and empty control construct. Student’s *t* test was used for statistical analysis

In our epigenetic work on *ZAR1*, we investigated the connection of promoter hypermethylation and repression of expression in the context of lung cancer. For promoter hypermethylation analysis, we chose bisulfite treatment and subsequent restriction analysis with *Taq*I enzyme (COBRA; combined bisulfite restriction analysis) and pyrosequencing for quantification (Additional file 1: Figure S1, target CpGs are shown). *ZAR1* expression was undetectable in various lung cancer cell lines as A427, A549 and H322, HTB171 and also in HeLa cervix cancer cells. H358 lung cancer cells however expressed *ZAR1* (Fig. 1a, b). In normal control tissues from an RNA panel, we found that *ZAR1* was detectable in the lung (set 100%), but relatively low (<10%) in breast, kidney, liver and heart. All lung cancer cell lines exhibited decreased *ZAR1* levels (<5%) vs. normal lung, except for H358, which was 15% (Fig. 1b). In lung cancer cell lines (A247, A549, HTB171, H322, H1299) and HEK cells, the *ZAR1* promoter was highly methylated as analysed by COBRA, in HeLa and H358 lung cancer cells partially methylated and in HCC-15 lung cancer cells unmethylated (Figs. 1c and 4a). The according quantification by pyrosequencing (Fig. 1d) confirmed the COBRA results and found methylation levels between 78 and 93% for A427, A549, HEK, HTB171 and H322 and partial promoter methylation of 50% for H358. An unmethylated sample (SC26, Fig. 3a) was used as a negative control (Fig. 1d). We then investigated *ZAR1* promoter hypermethylation by COBRA in a set of non-small cell lung cancer (NSCLC) and small cell lung cancer cells (SCLC) from cell lines and primary tumour samples (Additional file 2: Table S1). In SCLC cell lines, we observed ZAR1 promoter hypermethylation in 15 out of 20 samples (75%), and in the smaller set of NSCLC, five of six samples (83%) were methylated (Figs. 1, 2, 3c and 4a and Additional file 2: Table S1 and Additional file 3: Table S2). In primary tumour samples from SCLC, the *ZAR1* promoter was methylated in 5 out of 23 (22%) and in NSCLC, 16 out of 21 (76%) (Fig. 3 and Additional file 2: Table S1 and Additional file 3: Table S2). Control tissues of SCLC and NSCLC tumour samples were mostly unmethylated (11%; Fig. 3, Additional file 2: Table S1 and Additional file 3: Table S2). *ZAR1* promoter methylation increased from matching control tissues to tumour samples (LT1 to LN1, LT3 to LN3, LT39 to LN39, Fig. 3b).

Our findings showed a strong methylation of the *ZAR1* promoter, and we therefore aimed to test the epigenetic inactivation of *ZAR1* by reversal of promoter methylation. We chose lung cancer cell lines with *ZAR1* promoter hypermethylation for demethylation treatment in cell culture. 5-Aza-2’-deoxycytidine (Aza) is an inhibitor of DNA methyltransferases and demethylating agent. It was used to reverse promoter methylation and thereby verify the epigenetic inactivation of *ZAR1*. *ZAR1* methylated cell lines HEK, NSCLC cell lines A549, A427, H1299, H358, H322 and SCLC cell line HTB171 exhibited promoter demethylation with 5 or 10 μM Aza treatment, but unmethylated HCC-15 (NSCLC) remained unaffected (Fig. 4a). An in vitro methylated DNA was used as a positive control for.

Cancer cell lines (A427, A549, H322, HTB171 and HeLa) with *ZAR1* promoter hypermethylation (Figs. 1c, d and 4a) and missing *ZAR1* expression (Fig. 1a, b) were chosen for testing reexpression of *ZAR1* under demethylation treatment. All tested cancer cell lines showed endogenous reexpression of *ZAR1* with 5 μM Aza and further increased expression under 10 μM Aza in a semiquantitative PCR (Fig. 4b), which was consistent when quantified for 5 μM Aza by real-time PCR (Fig. 4c). Demethylation treatment restored *ZAR1* levels in H322 to almost normal lung expression levels (Fig. 4c). Coherently, the *ZAR1* promoter luciferase reporter construct was significantly silenced by in vitro methylation (ivm) compared to the unmethylated promoter or the empty control luciferease reporter construct (Fig. 4d).

### ZAR1 is a tumour suppressor in human cancer cell lines

**Fig. 5:**
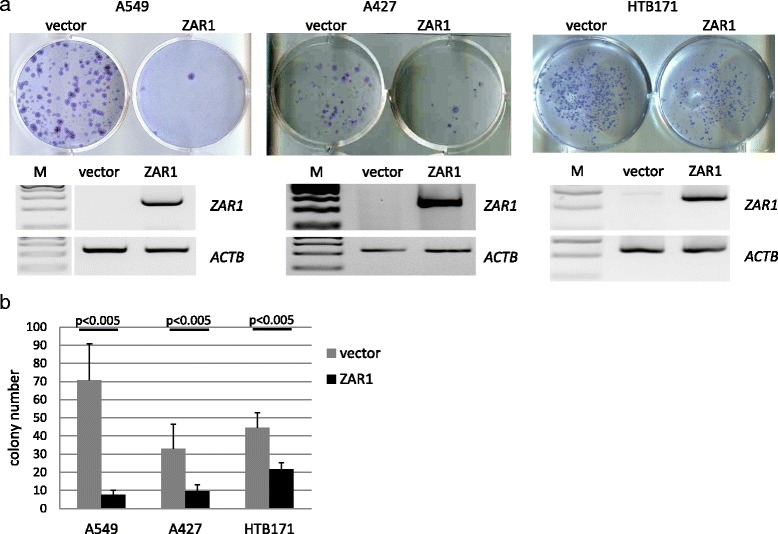
ZAR1 inhibits colony formation of lung cancer cell lines. ZAR1 was overexpressed in lung cancer cell lines A549, A427 and HTB171 and ZAR1 inhibited colony formation. Cells were transfected with ZAR1-EGFP or EGFP-vector control. Colonies were selected for 3 weeks with G418 and then Giemsa stained (**a**, *upper panel*). The according *ZAR1* overexpression was verified by semi-quantitative RT-PCR (**a**, *lower panel*). Inhibition of colony formation by ZAR1 was quantified and showed significant (Student’s *t* test) reduction in colony numbers (**b**)

**Fig. 6:**
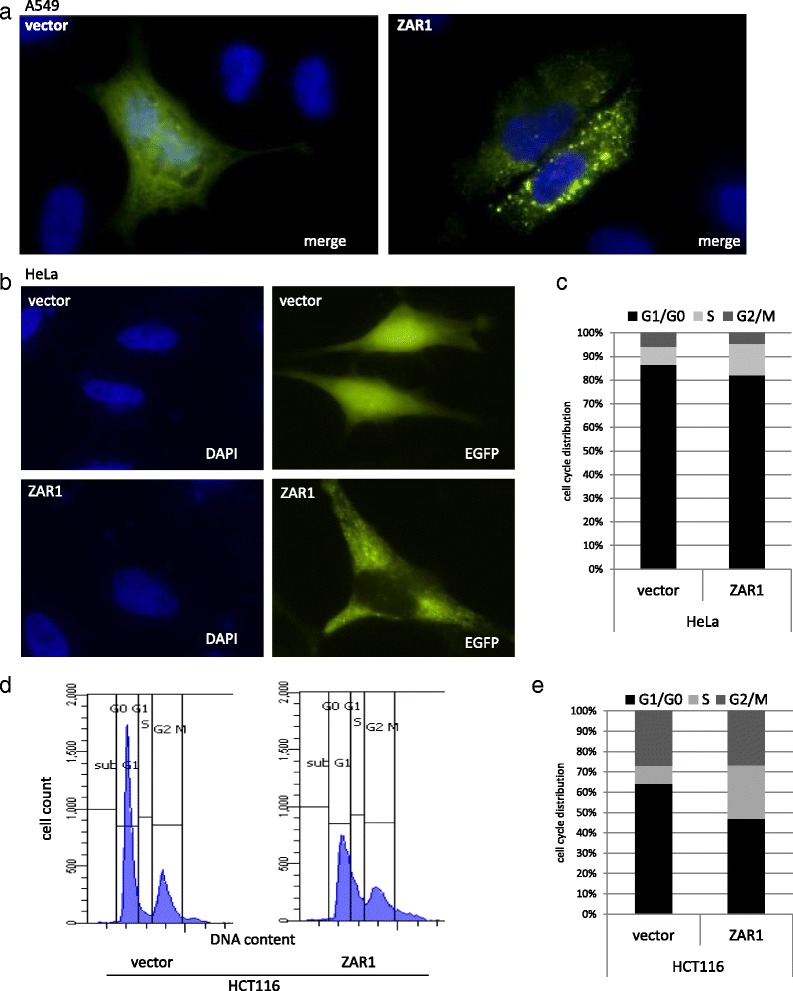
ZAR1 functions as a tumour suppressor in cancer cell lines through cell cycle arrest. **a**, **b** Localisation of overexpressed ZAR1 is exemplarily shown in A549 and HeLa with DAPI nuclear stain (×63 magnification). **c**–**e** Flow cytometry analysis found that overexpressed ZAR1 arrests the cell cycle in HeLa and HCT116 cancer cells. ZAR1 was overexpressed in cancer cells, cells were ethanol-fixed after 24 h (HeLa) and 48 h (HCT116) and propidium iodide staining was used to determine the DNA content for flow cytometry. The cell cycle distribution of HeLa and HCT116 cells upon ZAR1 reexpression is quantified (**c**, **e**), and gating is exemplarily depicted in HCT116 (**d**)

Based on our findings that *ZAR1* is epigenetically inactivated in lung cancer cell lines and primary lung cancer tumours, we aimed to test its tumour suppressive properties. Colony formation assays were performed in A549, A427 and HTB171 lung cancer cells that are epigenetically inactivated for *ZAR1* (Figs. 1 and 4). ZAR1 reexpression reduced colony numbers compared to vector control in A549 by 89%, in A427 by 71% and in HTB171 by 51% (Fig. 5a). This growth reduction by ZAR1 was significant for all three lung cancer cell lines when quantified (Fig. 5b). For expression control and determining ZAR1 localisation, we used EGFP tagged ZAR1. ZAR1 is found throughout the cytosol, but not within the nucleus, which is exemplarily shown for A549 and HeLa cells (Fig. 6a, b). We next questioned if the observed growth inhibition of lung cancer cells by reexpressed *ZAR1* could be due to increased apoptosis or arrest of cell cycle progression. We chose different cancer cells as A549 (lung cancer), HeLa (cervix carcinoma) and HCT116 (colon carcinoma) for reexpression of ZAR1. We performed flow cytometry analysis using propidium iodide staining to determine the nuclear DNA content. Flow cytometry in HeLa cells showed that ZAR1 overexpression increased the number of cells in S phase, but at the same time, no increase in mitosis was observed (Fig. 6c and Additional file 4: Figure S2A). Likewise, ZAR1 expression arrested HCT116 cells in S phase (Fig. 6d, e and Additional file 4: Figure S2C + D) as well as A549 cells (Additional file 4: Figure S2B).

In summary, we could show the epigenetic inactivation of *ZAR1* in lung cancer for the first time. Additionally, we characterised the tumour suppressive properties of ZAR1 in cancer cells. ZAR1 inhibited cancer cell growth by arresting cell cycle progression.

## Discussion

ZAR1 has been studied for a bit more than a decade and has been reported to be a maternal-effect gene critical for oocyte-to-embryo transition [10]. Subsequent reports found that the expression of *ZAR1* was not limited to the oocyte. In cattle [12], pig [13] and chicken [14], the *Zar1* expression pattern differs from mouse and *ZAR1* was not only expressed in ovary. *ZAR1* is also expressed in porcine and bovine brain and testis [13], additionally in bovine heart and muscle [12] and amongst others in chicken testis [14]. During the course of this project, it was reported that expression levels of rabbit *Zar1* are highest in lung tissue in comparison to other tissues, which supports our findings of strong *ZAR1* expression in human lung [21]. However, functionally, there is little known about ZAR1. ZAR1 female double-knockout mice are infertile due to developmental failure of their embryos [10]. In mouse, Cis-acting elements necessary for oocyte-specific gene expression were found in the *ZAR1* promoter [22]. Concerning xenopus Zar1, it has been said to bind to the 3’UTRs in maternal mRNAs through its zinc finger [20]. In our study, we aimed to establish ZAR1, which we found as a candidate from a 450 K methylation array, as an epigenetically inactivated tumour suppressor in human lung cancer.

In our study, we found that *ZAR1* was predominantly expressed in human lung, but relatively lowered in other tested tissues. This finding was in accordance with data we analysed from The GTEx Consortium Analysis Working Group [23], showing that the expression of *ZAR1* is not limited to oocytes in humans. In addition, we observed the absence of *ZAR1* expression in lung cancer cell lines. This observation prompted us to suspect an epigenetic regulation of *ZAR1* and a possible function of ZAR1 in cancer inhibition. Earlier reports found non-promoter hypermethylation of *ZAR1*; however, a functional consequence was not reported [15–19]. In contrast, we found promoter hypermethylation of *ZAR1* not only in lung cancer cell lines but also in primary lung cancer of the SCLC and NSCLC subtype. Furthermore, we could restore *ZAR1* expression in lung cancer cell lines pharmacologically by inhibition of DNA methyltransferases. Our analyses of *ZAR1* therefore define *ZAR1* as a heavily methylated CpG island carrying gene, which is epigenetically inactivated in lung cancer. It will be interesting to study the *ZAR1* hypermethylation together with other known lung cancer tumour suppressors, e.g. *RASSF1A* and *RASSF10* [24–28]. Defining a set of tumour suppressor that are specifically inactivated in lung cancer could serve as a tool for lung cancer detection in, e.g. liquid biopsies and as a cancer prognostic outcome predictor in the future. In larger sample sets of SCLC and NSCLC as well as lung cancer metastases, one could narrow the time point when *ZAR1* becomes hypermethylated and inactivated during lung carcinogenesis.

Interestingly, enhancer of zeste homolog 2 (EZH2), a part of the Polycomb Repressive Complex 2, was found to bind to the *ZAR1* promoter by ChIPseq as shown by the UCSC genome browser using the ENCODE project data (Additional file 5: Figure S3). EZH2 was said to be required for DNA methylation of EZH2-target promoters [29]. This observation supports the hypothesis that ZAR1 can be deliberately inactivated by DNA methylation in cancer. It is already well studied that methylated DNA in CpG island promoters is associated with the binding methylated DNA-binding proteins, which in turn recruit histone deacetylases to the region. This mechanism holds the promoter in a stably repressed state [30].

We found that overexpressed ZAR1 localised throughout the cytosol. Where endogenous ZAR1 is localised will be verified with later experiments when an appropriate antibody for ZAR1 is available. With this tool on-hand, we could not only study ZAR1 expression in primary cancer samples and correlate expression with *ZAR1* promoter methylation but also study interacting partners, which would give insight into its network and signalling pathways. This could elucidate its precise biological function in somatic lung cells, as to whether it controls proliferation, could induce apoptosis upon cellular stress and regulates mRNA translatability via binding to 3’UTRs [31]. In our present study, we further found that ZAR1 inhibited proliferation irrespective of the cancer type, as we observed ZAR1 blocked growth of three lung cancer cell lines, a cervix carcinoma and one colon cancer cell line. In detail, we found that ZAR1 interferes with S phase progression of the cell cycle. Further studies are needed to reveal through which pathways and by what partners ZAR1 inhibits cancer progression.

## Conclusions

Our findings give insight into the properties of ZAR1 in non-oocyte tissues and its tumour suppressive role in lung cancer. Together with the observed epigenetic inactivation of *ZAR1* in lung cancer and other cancer cell lines, we propose ZAR1 to be an epigenetically inactivated tumour suppressor in lung cancer. We demonstrated that ZAR1 could repress growth of cancer cell lines by inhibiting cell cycle progression. We therefore suggest that in the future, ZAR1 could serve as a hypermethylated biomarker for lung cancer detection and possibly could help differentiate between SCLC and NSCLC.

## Methods

### Analysis of the genomic and protein structure of ZAR1

The promoter region of *ZAR1* was analysed by CpG plot *http://www.ebi.ac.uk/Tools/seqstats/emboss_cpgplot/* and shows the existence of a 1.5 kb large CpG island. Primers for bisulfite-treated DNA were designed to bind only fully converted DNA and amplify the promoter region of *ZAR1*. The precise promoter region was chosen for CpG content and presence of according restriction enzymes for COBRA analysis. The size of the *ZAR1* COBRA PCR product is 186 bp (with *Taq*I site at 89). Additional file 1: Figure S1 shows an overview of the *ZAR1* gene and protein product, *ZAR1* CpG island, COBRA PCR product, primer positions, *Taq*I restriction site and pyrosequencing region.

### Cell lines, lung cancer tissues and controls

Lung cancer cell lines (A427, A549, H322, H358, H1299 and HCC15) were described previously [26]. SCLC cancer cell lines (CPC-N, DMS-79, H60, H69, H82, H187, H209, H510, H1092, H1184, H1339, H1963, H2171, H526, HCC-33, SCLC-21H, SCLC-24H, SCLC-86 M1) and HCT116 were obtained from Th. Stiewe. All lung cancer tissues were characterised previously [7, 25, 27]. All patients signed informed consent at initial clinical investigation. The study was approved by local ethic committees [7, 25, 27].

### DNA methylation analysis by COBRA and pyrosequencing

Two microgram genomic DNA (isolation by phenol-chloroform extraction) from tumour tissue, control tissue and cancer cell lines was bisulfite treated (12 μl 0.1 M hydroquinone, 208 μl 1.9 M sodium metabisulfite and pH 5.5 with NaOH) and incubated over night at 50 °C. DNA was purified using MSB Spin PCRapace (STRATEC Molecular), eluted in 50 μl H_2_O and incubated for 10 min with 5 μl 3 M NaOH at 37 °C. DNA was then precipitated with 100% ethanol and 7.5 M ammonium acetate and resolved in 30 μl 1 × TE buffer. Bisulfite treated DNA was subsequently used for 25 μl PCR reaction with *ZAR1* COBRA primers. The PCR product was digested with 0.5 μl of *Taq*I (Thermo Fisher Scientific) 1 h at 65 °C and resolved on 2% TBE gel together with mock control. Pyrosequencing was performed according to manufacturer’s protocol with PyroMark Q24 System (Qiagen). In vitro methylation of genomic/plasmid DNA for positive control in COBRA assay and luciferase promoter assay was performed using CpG methyltransferase *M.Sss*I (NEB) according to manufacturer’s protocol. Primers for COBRA analysis of the *ZAR1* promoter (186 bp) were upper primer GGAGAAGGAYGAAGAGGGGTTTTT and lower primer TCCCCCAAAACCRCCATAAAC. For pyrosequencing, the lower *ZAR1* primer was biotinylated. Fisher exact test was used for statistical analysis.

### RNA expression analysis

RNA was isolated using Isol-RNA lysis procedure (5 Prime). RNA was DNase (Thermo Fisher Scientific) treated and then reversely transcribed by MMLV (Promega). Semi-quantitative PCR was performed in Eppendorf Mastercycler using a standard protocol. Quantitative RT–PCR was performed in triplicate with SYBR select (Thermo Fisher Scientific) using Rotor-Gene 3000 (Qiagen). Normal human RNAs were obtained from Agilent Technologies. All other RNAs were isolated from cultured cancer cell lines. Primers for RT-PCR were upper primer CCTTCCTTCCTGGGCATGGAGTC and lower primer CGGAGTACTTGCGCTCAGGAGGA for *ACTB* (226 bp), upper primer AGCTGGGCAAGGAGCGGCTG and lower primer GGTGGGGCCGTTTAGGGTCCA for *ZAR1* (264 bp) and upper primer TGGAGAAGGCTGGGGCTCAT and lower primer GACCTTGGCCAGGGGTGCTA for GAPDH (176 bp). Student’s *t* test was used for statistical analysis.

### Cell culture, demethylation treatment (Aza) and transfection

Cell lines were grown in appropriate medium (DMEM or RPMI) supplemented with 10% FCS, and 1% penicillin/streptomycin under cell culture conditions (37 °C, 5% CO_2_). For 5-Aza-2’deoxycytidine (Aza) treatment cancer cell lines were split to 10% density, Aza was added with fresh medium on four consecutive days and RNA as well as DNA were isolated. For cell line transfection, we used Turbofect (Thermo Fisher Scientific), X-tremeGENE HP (Roche) or polyethylenimin (Sigma) with either 4 μg (6-well plates) or 10 μg (10 cm dishes) of ZAR1-pEGFP, EGFP vector, ZAR1-pRLnull or pRLnull empty.

### Colony formation and cell cycle analysis

For colony formation, assay cells were seeded at 10% density, transfected the following day. Selection with G418 (Biochrom) with 2.5 mg/ml (A549) and 0.25 mg/ml (A427, HTB171) started the following day and continued for 21 days. After 3 weeks, when visible colonies formed, cells were dried and stained with Giemsa (Sigma). Statistical analysis was performed using paired *T* test, two-tailed. Regarding flow cytometry analysis, cells were transfected with ZAR1-pEGFP or EGFP vector and cells were ethanol fixed after 24 or 48 h as indicated. The following day, fixed cells were PBS washed and treated with 50 μg/ml RNase A for 30 min at 37 °C. Subsequently, cells were stained with 50 μg/ml propidium iodide prior to measuring DNA content in FACSCantoII (BD Biosciences). FACSDiva Software (BD Biosciences) was used for measurement/gating to distinguish transfected fluorescent cells and to determine cells in G1/G0, S and G2/M phase of the cell cycle. ZAR1-EGFP overexpression is shown in HeLa and A549 cells. Therefore, cells were seeded in 6-well plates on glass slides and transfected the following day (see above). Cells were fixed with 3.7% formaldehyde at according time points, stained with DAPI (0.1 μg/ml in PBS, Sigma), embedded in anti-fading with Mowiol (Sigma) and analysed with Axio Observer Z1 (Zeiss) under ×63 magnification and by Volocity Software (Perkin Elmer).

### Plasmids and promoter reporter assay

*ZAR1* coding sequence was ordered from GeneArt AG (Regensburg, Germany) and cloned into pEGFP-C2 (Clontech). The *ZAR1* promoter (position +526 to −75 relative to transcriptional start site) was amplified from genomic DNA and cloned into pRL-null (Promega). Primers for cloning the *ZAR1* promoter were upper GGATCCCTGGGTGGTTCTCCATGCA and lower GAATTCGTAACCGTCCAGCACCTCGTCCCC (601 bp). All plasmids were controlled by sequencing, RT-PCR and immunofluorescence/Western blotting for the expression vector. For the promoter assay of *ZAR1*, the Dual-Luciferase Reporter Assay System (Promega) was used according to manufacturer’s protocol and HeLa cells were used. Student’s *t* test was used for statistical analysis.

## Additional files

Additional file 1: Figure S1. Schematic structure of the *ZAR1* gene/protein and CpG island. The *ZAR1* gene is shown with its four exons (encoding an 1275 nt transcript), and its protein (424 aa) structure with a C-terminal zinc finger (ZF). The *ZAR1* CpG island covers 1.5 kb of the promoter and the first exon. Black vertical lines represent single CpGs, and the restriction enzyme *Taq*I recognition site is marked (scissors) for COBRA methylation assay. Bent arrow indicates transcriptional start site (TSS). Horizontal arrows mark COBRA methylation analysis PCR product of 186 bp. Methylation analysis by pyrosequencing covers five CpGs (asterisk) within the COBRA analysed region. 1 kb standard is shown. (PDF 118 kb)

Additional file 2: Table S1. Supplemental Table 1. (XLSX 14 kb)

Additional file 3: Table S2. Supplemental Table 2. (XLSX 13 kb)

Additional file 4: Figure S2. ZAR1 overexpression blocks cell cycle progression of cancer cell lines. Cell lines HeLa, A549 and HCT116 were transfected with EGFP or ZAR1-EGFP, isolated after 24 h and fixed with ethanol. DNA content was measured by FACS CantoII using propidium iodide staining to determine cell cycle arrest. S phase alteration is marked in red. A–C Flow cytometry gating is shown for HeLa, A549 and HCT116 cancer cells and D shows quantification for HCT116. (PDF 345 kb)

Additional file 5: Figure S3. Genomic organisation of *ZAR1*. UCSC genome browser data revealed that transcription factors as EZH2 (arrowhead) bind to the promoter of *ZAR1* as analysed by ChIP-seq by the ENCODE project. EZH2 binding overlaps with the *ZAR1* CpG island promoter (green) and its first exon (blue). (PDF 576 kb)

## References

[1] Torre LA, Siegel RL, Jemal A (2016). Lung cancer statistics. Adv Exp Med Biol.

[2] Siegel R, Naishadham D, Jemal A (2013). Cancer statistics, 2013. CA Cancer J Clin.

[3] Ferlay J, Soerjomataram I, Dikshit R, Eser S, Mathers C, Rebelo M (2015). Cancer incidence and mortality worldwide: sources, methods and major patterns in GLOBOCAN 2012. Int J Cancer.

[4] Thun MJ, Hannan LM, Adams-Campbell LL, Boffetta P, Buring JE, Feskanich D (2008). Lung cancer occurrence in never-smokers: an analysis of 13 cohorts and 22 cancer registry studies. PLoS Med.

[5] Shi L, Zheng M, Hou J, Zhu B, Wang X. Regulatory roles of epigenetic modulators, modifiers and mediators in lung cancer. Semin Cancer Biol. 2016. Epub 2016/11/15. 10.1016/j.semcancer.2016.11.007 27840279

[6] Tan WL, Jain A, Takano A, Newell EW, Iyer NG, Lim WT (2016). Novel therapeutic targets on the horizon for lung cancer. Lancet Oncol.

[7] Dammann R, Li C, Yoon JH, Chin PL, Bates S, Pfeifer GP (2000). Epigenetic inactivation of a RAS association domain family protein from the lung tumour suppressor locus 3p21.3. Nat Genet.

[8] Kiehl S, Herkt SC, Richter AM, Fuhrmann L, El-Nikhely N, Seeger W (2014). ABCB4 is frequently epigenetically silenced in human cancers and inhibits tumor growth. Sci Rep.

[9] Kundu TK, Rao MR (1999). CpG islands in chromatin organization and gene expression. J Biochem.

[10] Wu X, Viveiros MM, Eppig JJ, Bai Y, Fitzpatrick SL, Matzuk MM (2003). Zygote arrest 1 (Zar1) is a novel maternal-effect gene critical for the oocyte-to-embryo transition. Nat Genet.

[11] Wu X, Wang P, Brown CA, Zilinski CA, Matzuk MM (2003). Zygote arrest 1 (Zar1) is an evolutionarily conserved gene expressed in vertebrate ovaries. Biol Reprod.

[12] Brevini TA, Cillo F, Colleoni S, Lazzari G, Galli C, Gandolfi F (2004). Expression pattern of the maternal factor zygote arrest 1 (Zar1) in bovine tissues, oocytes, and embryos. Mol Reprod Dev.

[13] Uzbekova S, Roy-Sabau M, Dalbies-Tran R, Perreau C, Papillier P, Mompart F (2006). Zygote arrest 1 gene in pig, cattle and human: evidence of different transcript variants in male and female germ cells. Reprod Biol Endocrinol.

[14] Michailidis G, Argiriou A, Avdi M (2010). Expression of chicken zygote arrest 1 (Zar1) and Zar1-like genes during sexual maturation and embryogenesis. Vet Res Commun.

[15] Shinojima Y, Terui T, Hara H, Kimura M, Igarashi J, Wang X (2010). Identification and analysis of an early diagnostic marker for malignant melanoma: ZAR1 intra-genic differential methylation. J Dermatol Sci.

[16] Watanabe T, Yachi K, Ohta T, Fukushima T, Yoshino A, Katayama Y (2010). Aberrant hypermethylation of non-promoter zygote arrest 1 (ZAR1) in human brain tumors. Neurol Med Chir (Tokyo).

[17] Sugito K, Kawashima H, Yoshizawa S, Uekusa S, Hoshi R, Furuya T (2013). Non-promoter DNA hypermethylation of Zygote Arrest 1 (ZAR1) in neuroblastomas. J Pediatr Surg.

[18] Hasegawa R, Fujiwara K, Obinata D, Kawashima H, Shinojima Y, Igarashi J (2015). Identification of frequent differentially methylated region in sporadic bladder cancers. Urol Int.

[19] Takagi K, Fujiwara K, Takayama T, Mamiya T, Soma M, Nagase H (2013). DNA hypermethylation of zygote arrest 1 (ZAR1) in hepatitis C virus positive related hepatocellular carcinoma. Springerplus.

[20] Yamamoto TM, Cook JM, Kotter CV, Khat T, Silva KD, Ferreyros M (2013). Zar1 represses translation in Xenopus oocytes and binds to the TCS in maternal mRNAs with different characteristics than Zar2. Biochim Biophys Acta.

[21] Wang D, Xie SY, Zhang W, Sun CX, Huang T, Wang AS (2017). Cloning and expression analysis of zygote arrest 1 (Zar1) in New Zealand white rabbits. J Genet.

[22] Tsunemoto K, Anzai M, Matsuoka T, Tokoro M, Shin SW, Amano T (2008). Cis-acting elements (E-box and NBE) in the promoter region of three maternal genes (Histone H1oo, Nucleoplasmin 2, and Zygote Arrest 1) are required for oocyte-specific gene expression in the mouse. Mol Reprod Dev.

[23] Consortium GT (2013). The Genotype-Tissue Expression (GTEx) project. Nat Genet.

[24] Richter AM, Walesch SK, Wurl P, Taubert H, Dammann RH (2012). The tumor suppressor RASSF10 is upregulated upon contact inhibition and frequently epigenetically silenced in cancer. Oncogenesis.

[25] Dammann R, Takahashi T, Pfeifer GP (2001). The CpG island of the novel tumor suppressor gene RASSF1A is intensely methylated in primary small cell lung carcinomas. Oncogene.

[26] Dammann R, Strunnikova M, Schagdarsurengin U, Rastetter M, Papritz M, Hattenhorst UE (2005). CpG island methylation and expression of tumour-associated genes in lung carcinoma. Eur J Cancer.

[27] Helmbold P, Lahtz C, Herpel E, Schnabel PA, Dammann RH (2009). Frequent hypermethylation of RASSF1A tumour suppressor gene promoter and presence of Merkel cell polyomavirus in small cell lung cancer. Eur J Cancer.

[28] Richter AM, Walesch SK, Dammann RH. Aberrant Promoter Methylation of the Tumour Suppressor RASSF10 and Its Growth Inhibitory Function in Breast Cancer. Cancers (Basel). 2016;8(3). Epub 2016/03/02. 10.3390/cancers8030026 PMC4810110 26927176

[29] Vire E, Brenner C, Deplus R, Blanchon L, Fraga M, Didelot C (2006). The Polycomb group protein EZH2 directly controls DNA methylation. Nature.

[30] Jones PA (2012). Functions of DNA methylation: islands, start sites, gene bodies and beyond. Nat Rev Genet.

[31] Szostak E, Gebauer F (2013). Translational control by 3′-UTR-binding proteins. Brief Funct Genomics.

