# Supplementary material for: ZAR1 is a novel epigenetically inactivated tumour suppressor in lung cancer
Source: Clin Epigenetics. 2017 Jun 2;9:60. doi: 10.1186/s13148-017-0360-4 (PMC5457737; doi:10.1186/s13148-017-0360-4)
Supplement: Supplementary file 1 — Schematic structure of the ZAR1 gene/protein and CpG island. The ZAR1 gene is shown with its four exons (encoding an 1275 nt transcript), and its protein (424 aa) structure with a C-terminal zinc finger (ZF). The ZAR1 CpG island covers 1.5 kb of the promoter and the first exon. Black vertical lines represent single CpGs, and the restriction enzyme TaqI recognition site is marked (scissors) for COBRA methylation assay. Bent arrow indicates transcriptional start site (TSS). Horizontal arrows mark COBRA methylation analysis PCR product of 186 bp. Methylation analysis by pyrosequencing covers five CpGs (asterisk) within the COBRA analysed region. 1 kb standard is shown. (PDF 118 kb) [file 13148_2017_360_MOESM1_ESM.pdf]

Figure S1:

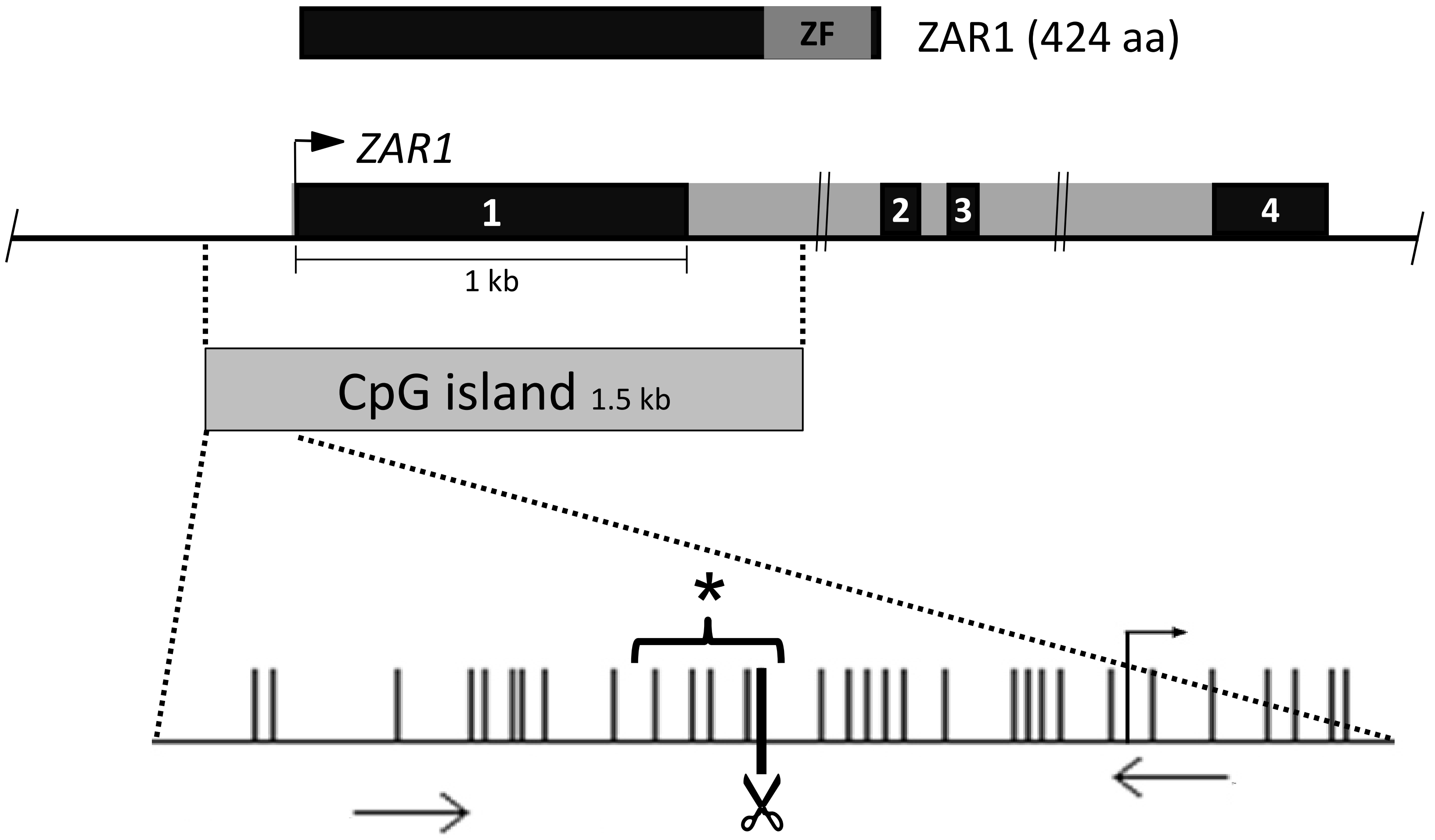

Figure S1. Schematic structure of the ZAR1 gene/protein and CpG island The *ZAR1* gene is shown with its four exons (encoding an 1275 nt transcript) and its protein (424 aa) structure with a C-terminal zinc finger (ZF). The *ZAR1* CpG island covers 1.5 kb of the promoter and the first exon. Black vertical lines represent single CpGs and the restriction enzyme *TaqI* recognition site is marked (scissors) for COBRA methylation assay. Bent arrow indicates transcriptional start site (TSS). Horizontal arrows mark COBRA methylation analysis PCR product of 186 bp. Methylation analysis by pyrosequencing covers five CpGs (asterisk) within the COBRA analysed region. 1 kb standard is shown.
