# Supplementary material for: ZAR1 is a novel epigenetically inactivated tumour suppressor in lung cancer
Source: Clin Epigenetics. 2017 Jun 2;9:60. doi: 10.1186/s13148-017-0360-4 (PMC5457737; doi:10.1186/s13148-017-0360-4)
Supplement: Supplementary file 4 — ZAR1 overexpression blocks cell cycle progression of cancer cell lines. Cell lines HeLa, A549 and HCT116 were transfected with EGFP or ZAR1-EGFP, isolated after 24 h and fixed with ethanol. DNA content was measured by FACS CantoII using propidium iodide staining to determine cell cycle arrest. S phase alteration is marked in red. A–C Flow cytometry gating is shown for HeLa, A549 and HCT116 cancer cells and D shows quantification for HCT116. (PDF 345 kb) [file 13148_2017_360_MOESM4_ESM.pdf]

Figure S2:

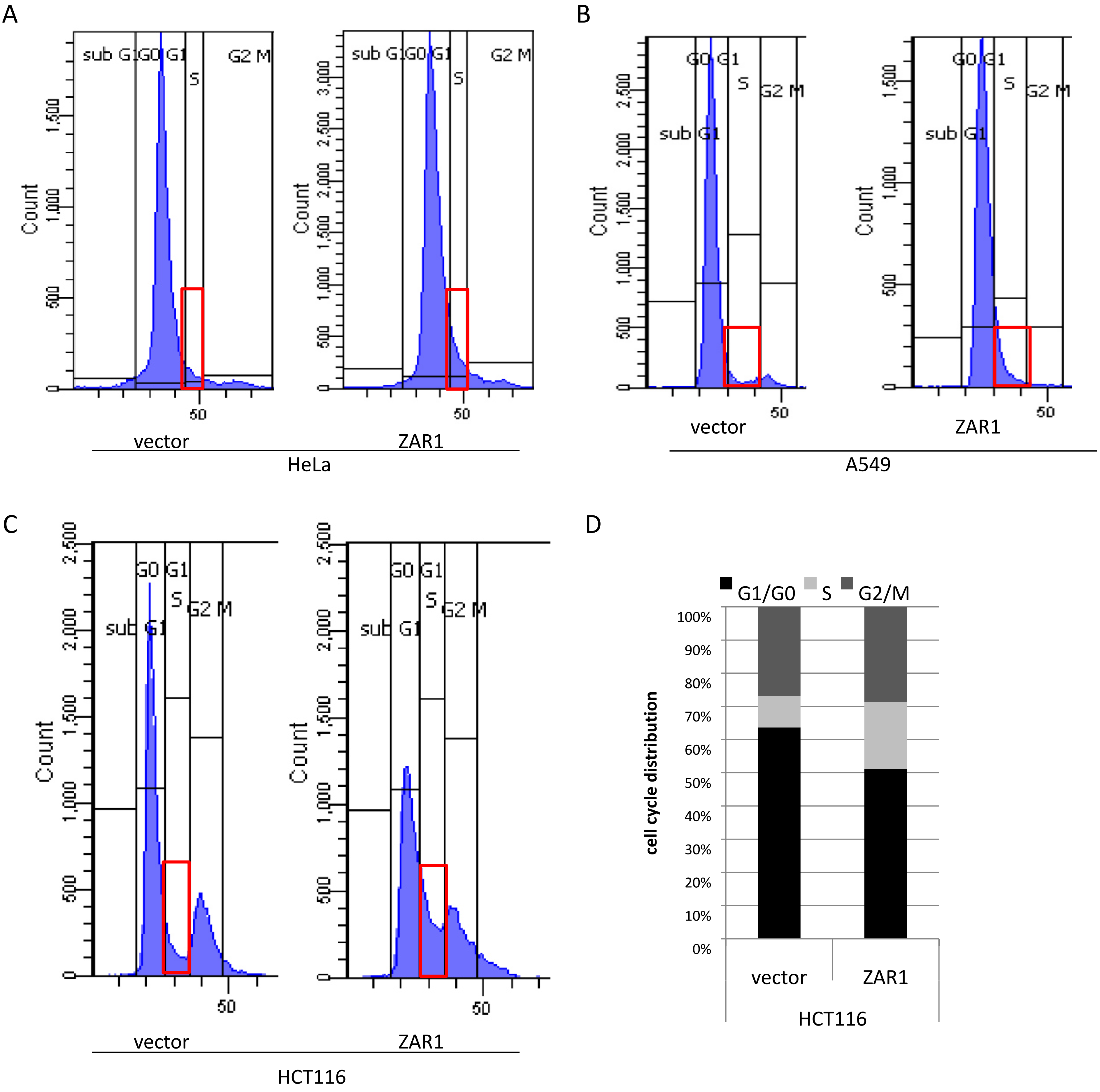

ZAR1 overexpression blocks cell cycle progression of cancer cell lines. Cell lines HeLa, A549 and HCT116 were transfected with EGFP or ZAR1EGFP, isolated after 24h and fixed with ethanol. DNA content was measured by FACS CantoII using propidium iodide staining to determine cell cycle arrest. S Phase alteration is marked in red. A-C) Flow cytometry gating is shown for HeLa, A549 and HCT116 cancer cells and D) shows quantification for HCT116.
