# Supplementary material for: ZAR1 is a novel epigenetically inactivated tumour suppressor in lung cancer
Source: Clin Epigenetics. 2017 Jun 2;9:60. doi: 10.1186/s13148-017-0360-4 (PMC5457737; doi:10.1186/s13148-017-0360-4)
Supplement: Supplementary file 5 — Genomic organisation of ZAR1. UCSC genome browser data revealed that transcription factors as EZH2 (arrowhead) bind to the promoter of ZAR1 as analysed by ChIP-seq by the ENCODE project. EZH2 binding overlaps with the ZAR1 CpG island promoter (green) and its first exon (blue). (PDF 576 kb) [file 13148_2017_360_MOESM5_ESM.pdf]

Figure S3:

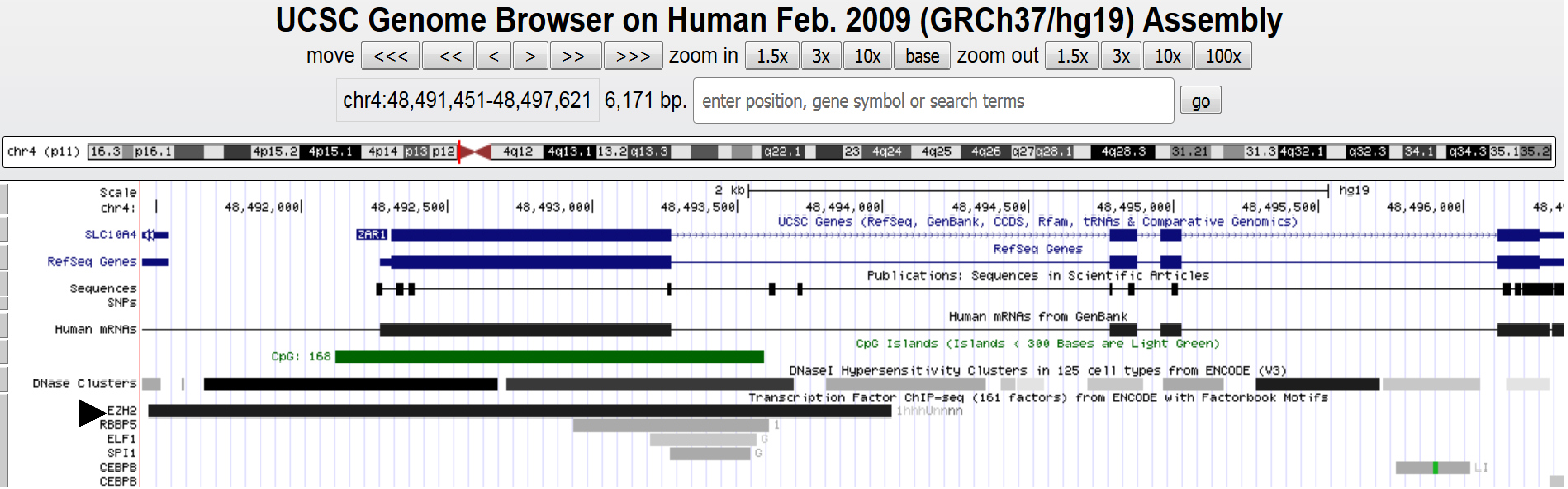

Genomic organisation of ZAR1. UCSC genome browser data revealed that transcription factors as EZH2 (arrowhead) bind to the promoter of ZAR1 as analysed by ChIP-seq by the ENCODE project. EZH2 binding overlaps with the ZAR1 CpG island promoter (green) and its first exon (blue).
